# Computerized Cognitive Enrichment as a Conservation Engagement Tool: Shifting Visitor Perceptions of the Endangered Hawaiian Monk Seal (*Neomonachus schauinslandi*)

**DOI:** 10.3390/ani16213372

**Published:** 2026-10-26

**Authors:** Amber M. Ramos, Danni Robinette, Shivang Sharma, Kelley A. Winship

**Affiliations:** 1 Sea Life Park, Hawaiʻi 41-202 Kalanianaole Highway #7, Waimanalo, HI 96795, USA; 2 Department of Natural Sciences, Hawaiʻi Pacific University, 1 Aloha Tower Dr, Honolulu, HI 96813, USA; 3 Animal Computerized Enrichment, Junction, TX 76849, USA

**Keywords:** Hawaiian monk seal, animal-computer interaction, cognitive enrichment, conservation attitudes, zoo and aquarium visitors, human–animal interaction

## Abstract

Public viewing of cognitive enrichment may offer opportunities to connect visitors with endangered species through observation of animal learning and problem-solving. We examined visitor responses to a narrated demonstration involving a Hawaiian monk seal (*Neomonachus schauinslandi*) using the Enclosure Video Enrichment system at Sea Life Park Hawaiʻi. In this exploratory single-group study, 104 adults completed questionnaires before and immediately after the session. Six agreement items assessed perceptions of cognition, connection, caring, and protection; two questions addressed endangered status and the ability to learn a computer game. Paired responses were analyzed using Wilcoxon signed-rank and exact McNemar tests, with Bonferroni adjustment across eight comparisons. Ratings increased significantly for five agreement items after adjustment, with the largest mean changes in connection and perceived problem-solving. Protection ratings were high initially and did not increase significantly after adjustment. Correct computer-game responses increased while endangered-status knowledge remained high. The single-group design limits causal interpretation and attribution to computerized enrichment specifically. These findings highlight public enrichment demonstrations as a promising setting for conservation education. Future studies should assess whether changes in perceptions and connection persist and contribute to behaviors that support endangered species recovery.

## Simple Summary

Computerized tasks provide animals opportunities to make choices, solve problems, and use skills that may not be obvious to zoo and aquarium visitors. This study examined visitor responses after observing Kekoa, a Hawaiian monk seal, interact with a computerized, cognitive enrichment system at Sea Life Park, Hawaiʻi. A total of 104 adults completed questionnaires before and after the session. Post-session, visitors rated monk seals as smarter and more capable of learning and solving problems. They also reported feeling more connected to monk seals. Most participants already knew that Hawaiian monk seals are endangered and strongly supported their protection, so these responses changed little. These results show that a public enrichment session can help visitors better understand an endangered animal and feel more connected to its species. Computerized cognitive enrichment may provide a useful link between animal care and conservation education.

## 1. Introduction

### 1.1. Conservation Context for the Hawaiian Monk Seal

The Hawaiian monk seal (*Neomonachus schauinslandi*), known in ʻōlelo Hawaiʻi as ʻīlio holo i ka uaua, is endemic to the Hawaiian archipelago and remains endangered despite decades of recovery work [1,2]. NOAA Fisheries estimates that approximately 1600 seals remain, including nearly 1200 in Papahānaumokuākea in the Northwestern Hawaiian Islands and about 400 in the main Hawaiian Islands [2]. The population has increased gradually since 2013, reversing a prolonged decline [3,4], but it remains substantially below its historical abundance [5]. This combination of modest recovery and continued vulnerability makes protection of individual seals, particularly juveniles and reproductive females, an ongoing conservation priority [2,6].

In the remote Northwestern Hawaiian Islands, limited food availability and historically low juvenile survival have contributed to population declines, while changing habitat conditions and marine debris create additional challenges [2,6]. In the main Hawaiian Islands, a growing population of monk seals increasingly shares beaches and coastal waters with residents, tourists, fishers, and domestic animals [7]. Here, interactions involving fishing gear, disturbance of resting seals and mothers with pups, and disease can threaten survival [2,6]. Toxoplasmosis is a particularly concerning disease associated with land-based sources of contamination; in August 2026, NOAA reported that it had caused the death of juvenile seal RU02 on Oʻahu [8,9,10]. These varied threats require conservation measures that address both ecological pressures and risks arising from human activity [11,12,13,14,15,16].

Recent deaths of young monk seals across the main Hawaiian islands reflect ongoing conservation challenges. In August 2026, responders found approximately three-month-old pup RV36 deceased and entangled in an unattended fishing net off Oʻahu [10,17,18]. NOAA also confirmed the death of two-year-old RT68 on Oʻahu that month, without establishing a cause in its initial announcement [8,19]. On Molokaʻi, one-year-old female RU68 was found dead in August 2026, and NOAA reported in September that preliminary evidence suggested blunt-force trauma [20].

Reported incidents of deliberate disturbance also illustrate the relevance of public understanding. In May 2026, a visitor on Maui was filmed throwing a rock at a monk seal; his attorney later stated that he had mistaken the animal for an aggressive sea lion, a species not found in Hawaii. [21,22,23]. In August 2026, witnesses reported that two people approached a monk seal pup at Diamond Head Beach on Oʻahu and threw a rock at it [24]. Such encounters reinforce the importance of recognizing monk seals, understanding their vulnerability, and responding to them with concern. Public knowledge, emotional connection, and empathy are therefore relevant conservation goals and provide context for examining visitor responses to individual animals.

### 1.2. Zoo Experiences, Animal Cognition, and Visitor Perceptions

Zoos and aquariums provide opportunities for visitors to observe animals, learn about biodiversity, and encounter conservation messages [25]. Studies have examined immediate changes in biodiversity understanding and conservation-relevant attitudes following zoo visits [26,27,28,29,30,31,32,33]. Moss and colleagues used matched pre- and post-visit measurements across 26 zoos and aquariums and found significant increases in visitors’ understanding of biodiversity and knowledge of actions they could take to protect it [29]. Visitor learning, feelings of connection, behavioral intentions, and actual behavior represent distinct outcomes through which these experiences can be evaluated [26,31,34]. Examining responses to specific activities can help identify their contribution to the broader visitor experience. The present study extends this research by examining immediate changes in perceptions of monk seal cognition, feelings of connection, and conservation-relevant attitudes following a public cognitive-enrichment demonstration.

Visitor responses may depend on how visable and active the aniamals are [35,36,37]. Watching animals solve problems may help visitors recognize their cognitive abilities. However, visitors’ responses may also be influenced by animal activity, trainer presence, narration, ot the novelty of the apparatus, rather than by the technology alone [38,39,40,41,42,43,44]. Studies of orangutans using interactive systems describe visitor interest and empathetic responses [38,39,40], whereas a comparison of videos of sun bears engaging with computerized versus conventional enrichment found no clear advantage of computerized tasks [42]. Studies such as these provide a foundation for investigating public cognitive-enrichment demonstrations as opportunities for visitor learning and connection with animals.

Cognitive enrichment involves opportunities for challenge, problem-solving, and choice [45,46,47,48,49]. Animal–computer interaction provides one method of delivering such opportunities [50,51,52,53,54]. The Enclosure Video Enrichment (EVE) system was developed for marine mammals, with studies of its use, welfare associations, and gameplay in California sea lions *(Zalophus californianus*) providing a welfare and husbandry rationale for its use [55,56,57,58,59] and a basis for exploring its educational potential for visitors. Recent work has also emphasized that studying marine mammal cognition can contribute to conservation by clarifying how animals acquire information, respond to environmental change, and adjust their behavior [60].

### 1.3. Present Study and Research Questions

We used a one-group, matched pre–post questionnaire design to describe immediate differences in visitors’ reported beliefs about monk seal cognition, personal connection, conservation-relevant attitudes, and responses to two factual questions. Visitors completed the same instrument before and after observing a Hawaiian monk seal in a narrated public EVE demonstration. The study was intended as an initial, naturalistic case evaluation, not as a controlled test of EVE-specific causality.

We expected higher post-session ratings of cognition and connection and examined the extent to which high baseline responses limited changes in protection-related attitudes. The question concerning whether monk seals can learn to play a computer game was treated as recognition of the behavior directly demonstrated, not as an independent measure of broad conservation knowledge. We did not measure behavioral intention, observed conservation action, or durability of any response.

## 2. Materials and Methods

### 2.1. Study Site and Animal

Kekoa (NOAA permanent identification number KE-18) is a Hawaiian monk seal residing at Sea Life Park Hawaiʻi on Oʻahu [61]. Before returning to his permanent home at Sea Life Park Hawaiʻi, he participated in auditory, physiological, and prey-capture research at the Long Marine Laboratory, University of California, Santa Cruz [62,63,64,65,66,67].

Kekoa was born in 2002 on Hōlanikū (Kure Atoll), the northwesternmost atoll in the Hawaiian archipelago and part of Papahānaumokuākea Marine National Monument. In 2010, he began exhibiting persistent single-male aggression toward younger seals [68]. These interactions can involve biting, restraining, and holding juvenile seals underwater, potentially resulting in serious injury or death [68]. Following repeated incidents, NOAA recommended euthanasia to prevent further harm to the wild population. The field team was unable to locate Kekoa and therefore could not carry out the procedure. Permits were subsequently obtained for his placement in professional care.

Kekoa was transferred to the Long Marine Laboratory, where he remained for more than nine years before returning to Hawaiʻi in 2021. He is housed at Sea Life Park Hawaiʻi under National Marine Fisheries Service permit 22851, issued pursuant to the Endangered Species Act and Marine Mammal Protection Act. He was 24 years old at the time of data collection.

Kekoa’s daily management includes feeding, rest, enrichment, and training sessions addressing routine husbandry, maintenance of established behaviors, acquisition of new behaviors, and educational presentations. Computerized cognitive enrichment forms part of this management program. Session structure and duration varied according to Kekoa’s behavior and the operational needs of animal care staff.

The human subjects component of this study was reviewed and approved by the Institutional Review Board (IRB) of Hawaiʻi Pacific University under an exempt review category. The cognitive enrichment activity was conducted under Sea Life Park Hawaiʻi’s Institutional Animal Care and Use Committee (IACUC) protocol #KW2026. Under the approved IACUC protocol, participation was voluntary, and Kekoa could decline participation or end a session at any time. Reinforcement included food and social interaction. Animal care staff occasionally observed him continuing to interact with the EVE system after declining offered food reinforcement.

For the present study, visitors observed Kekoa operating the EVE system during a public demonstration. The session combined his behavior, trainer interaction, food reinforcement, game performance, and scripted narration and was evaluated as a single visitor experience. Questionnaires administered before and immediately after the demonstration assessed visitors’ perceptions of monk seal cognition, feelings of connection, and conservation-relevant attitudes.

### 2.2. The Enclosure Video Enrichment (EVE) System

Participants observed a session with the Enclosure Video Enrichment (EVE) system, a computerized cognitive enrichment system developed at the US Navy Marine Mammal Program and described by Winship and colleagues [55]. A monitor housed within a mobile cart presents the visual stimuli. In the novel version, Kekoa controls an on-screen blue (HEX code #0808ff; rgba(8, 8, 255)) circular cursor by pressing four arcade buttons with his muzzle. Correct responses produce an audible tone and are followed by food reinforcement and praise from trainers positioned beside him.

Kekoa’s game catalog includes an introductory task of navigating a cursor to a target (based on Winship et al., 2023 [55]), chase tasks with one or multiple moving targets, maze navigation [57], a chase task with a human-controlled second player, and a three-dimensional navigation task. Additional games are being developed and playtested.

Kekoa’s engagement with EVE has been measured separately but was not an outcome of this study. Animal care staff reported that he generally participated without prompting and sometimes continued playing after declining offered food reinforcement. These observations are included as context and were not analyzed as study data.

### 2.3. Participants and Recruitment

Before each scheduled session, a researcher used a microphone to invite visitors aged 18 years or older to observe the demonstration and complete a two-part questionnaire. Participation was voluntary. Visitors who approached the researcher received a clipboard with the consent document and a pre-coded questionnaire (Part 1 on the front, Part 2 on the reverse). Recruitment produced a self-selected sample of adults in the proximity of the habitat during scheduled sessions. The approved protocol restricted participation to adults. Questionnaires completed by or on behalf of visitors under 18 years of age were excluded before analysis (see Table 1).

### 2.4. Questionnaire Instrument

The questionnaire consisted of two parts that included the same agreement and knowledge items. Participants rated six statements using a five-point Likert type scale [69] ranging from 1 (strongly disagree) to 5 (strongly agree): “Monk seals are smart animals,” “Monk seals can solve problems,” “Monk seals can learn new things,” “I feel connected to monk seals,” “I care what happens to monk seals,” and “It is important to protect monk seals.” Participants also answered two true-or-false knowledge questions: “Are Hawaiian monk seals endangered?” and “Can Hawaiian monk seals learn to play a computer game?”

The first three agreement statements assessed participants’ beliefs about the cognitive abilities of Hawaiian monk seals. The remaining three assessed emotional connection and attitudes toward conservation. Part 1 also collected background information, including confirmation that participants were at least 18 years old, their home location, whether they had previously visited Sea Life Park, and whether they were aware of Hawaiian monk seals before their visit. Part 2 repeated the six agreement statements and two knowledge questions using identical wording.

### 2.5. Procedure

Each participant’s pre- and post-session responses were linked using a date-letter code without identifying information. During the pre-session questionnaire, Kekoa was visible and typically swimming or resting; the EVE apparatus was brought into the habitat afterward. A scripted narration accompanied the EVE demonstration, and trainers provided cues, feedback, and reinforcement. All participants completed the questionnaire in the same order; no control condition, randomized allocation, or counterbalanced crossover was used.

Participants watched the session from the public viewing area (see Figure 1) and completed Part 2 immediately afterward. Each questionnaire took approximately two minutes, and the full procedure took seven to eight minutes. Participants received a debriefing form after both questionnaires were collected, and investigators remained available to answer questions. Data were collected on 12 days in summer 2026, with one or two sessions held each day. The number and timing of data-collection days reflected the availability of Kekoa, the training staff, as well as the park’s other operational and animal care constraints.

The study evaluated an existing visitor experience using a within-participant pre–post design. A control condition was not included because the questionnaire specifically asked about gameplay, potentially raising expectations and disappointing guests who learned about the activity but could not observe it. This design assessed immediate changes following the complete demonstration but could not separate the effects of gameplay from other components of the experience.

### 2.6. Data Handling and Exclusion

All collected responses were combined into a single data set with one row per participant. Participant-level exclusions were made when adult status could not be confirmed or field notes identified a protocol deviation (such as completing Part 2 prior to EVE presentation). Table 1 provides the number of records retained or removed and the reason for each exclusion. Records with missing items remained in the dataset, but each paired comparison included only participants who answered that item in both Part 1 and Part 2. Blank or unclear responses were treated as missing.

### 2.7. Data Analysis

Analyses were conducted using R statistical software (Version 4.6.0) [70] with available paired responses for each item. Two-sided Wilcoxon signed-rank tests assessed changes in the six agreement items, while exact McNemar tests compared incorrect-to-correct and correct-to-incorrect changes in the two knowledge items. Unadjusted and Bonferroni-adjusted *p* values were reported across these eight comparisons, with adjusted *p* < 0.05 indicating statistical significance.

Agreement ratings were summarized by mean paired changes and counts of increased, unchanged, and decreased responses. Matched-pairs rank-biserial correlations described the balance of upward and downward changes: (W^+^ − W^−^)/(W^+^ + W^−^), where W^+^ and W^−^ are positive and negative signed-rank sums excluding zero differences. These coefficients therefore describe responses that changed, rather than the full sample. The 95% confidence intervals for mean changes and rank-biserial correlations were calculated using the percentile bootstrap method with 3,000 participant-level paired resamples. Exploratory Spearman correlations assessed whether participants with higher pre-session ratings also tended to give higher post-session ratings.

## 3. Results

### 3.1. Questionnaire Records and Analytic Sample

Of the 146 questionnaires received, 140 yielded matched pre- and post-session records. After age and protocol-related exclusions, the final analytic sample comprised 104 adults (Table 1).

The final sample included 104 adults. Analyses used available paired responses for each item, resulting in sample sizes of 103–104 for agreement items, 103 for endangered-status knowledge, and 98 for the computer-game question. One unclear post-session response to the “smart animals” item was treated as missing, leaving 103 complete pairs for that item.

### 3.2. Change in Beliefs About Monk Seal Cognition and Conservation Attitudes

Average ratings increased after the demonstration for all six agreement items (Table 2, Figure 2). Five increases remained statistically significant after applying the Bonferroni correction for eight comparisons (all unadjusted *p* < 0.001). The increase in protection was not statistically significant after correction (unadjusted *p* = 0.016; adjusted *p* = 0.132). The large rank-biserial effect sizes describe only responses that changed, so they should be considered alongside the many responses that stayed the same.

Mean ratings increased by 0.85 points for feeling connected, 0.80 points for problem-solving, and 0.11 points for protection. High initial protection ratings likely left little room for improvement. Table 2 shows how many ratings increased, decreased, or stayed the same. Positive Spearman correlations indicate that respondents who gave higher ratings before the demonstration generally also gave higher ratings afterward, although the strength of this pattern varied across items. Table 2 provides the 95% confidence intervals for the mean changes.

### 3.3. Responses to Knowledge Questions

Correct endangered-status responses were already high at baseline (96.1%) and changed little (99.0%; *n* = 103, exact McNemar *p* = 0.375). Correct computer-game responses increased from 48.0% to 96.9% (*n* = 98; exact McNemar *p* < 0.001). The computer-game question primarily assessed immediate comprehension of the demonstrated behavior and cannot by itself establish generalized or lasting conservation knowledge.

For the computer-game item, 49 participants changed from an incorrect to a correct answer and one changed in the opposite direction. Complete-pair analyses retained all valid responses; a suspected marking error was not removed retrospectively. The computer-game item had four missing pre-session and two missing post-session responses, leaving 98 complete pairs. No inference about long-term retention can be made.

### 3.4. Exploratory Comparisons by Visitor Origin and Prior Visit

Home location and prior visit to Sea Life Park were recorded in the pre-session questionnaire, and comparisons using these variables were conducted after the primary analysis rather than being specified in advance. When grouped by region as Hawaiʻi residents, continental United States residents, or international residents, we did not find differences in change on any of the six agreement items, with all *p* values above 0.13. Prior visit to Sea Life Park was similarly unrelated to change.

## 4. Discussion

The public EVE demonstration was associated with positive changes in visitors’ perceptions of Hawaiian monk seal cognition and their feelings of connection to the species. Ratings of cognitive ability, connection, and caring increased following the session, and these five items remained statistically significant after adjustment for multiple comparisons. Perceived problem-solving and personal connection showed the largest mean increases. These findings extend research on visitor responses to animal cognition to an endangered marine mammal and identify public cognitive enrichment as a promising setting for conservation education.

Cognitive enrichment provides opportunities for animals to learn, solve problems, and exercise choice [45,46,47,48,71,72,73,74,75]. EVE applies these principles within marine mammal care, with previous studies examining its use and welfare associations in California sea lions [55,56,57]. In the present study, Kekoa’s operation of the system made learned, task-directed behavior visible to visitors during an established animal-care activity. This offers a practical opportunity to discuss an animal’s abilities through direct observation. Educational interpretation can accompany enrichment while participation and session decisions remain guided by the animal’s behavior and care needs.

The pattern of visitor responses is consistent with research linking animal activity, interactive technology, and public scientific research with visitor interest and perceptions [35,36,37,38,39,40,43,44]. These experiences bring attention to behaviors that may be less apparent during routine viewing. Their value also depends on the wider presentation [76]. Perdue and Robinson [42], for example, found similar visitor outcomes studying visitor perception of traditional versus cognitive enrichment. In the present setting, gameplay, trainer interaction, food reinforcement, and narration formed a combined experience through which visitors encountered Kekoa’s behavior.

The increase in reported connection is particularly relevant to conservation education. Sixty-four of 103 respondents rated their connection higher after the session, while 38 reported no change and one reported a decrease. Research on conservation caring links connection to wildlife with willingness to support species conservation [26,32,33]. The present findings suggest that a brief encounter centered on an individual monk seal may provide an opportunity for visitors to relate more personally to the species. Perceptions of cognition, emotional connection, and conservation behavior remain distinct outcomes; the observed changes provide a basis for examining how these outcomes relate in subsequent studies.

Visitors entered the demonstration with strong support for monk seal protection and high awareness of the species’ endangered status. These favorable starting points left limited room for further increases. The small change in protection ratings did not remain statistically significant after adjustment for eight comparisons, whereas the increases in cognition, connection, and caring did. This pattern suggests that public enrichment may have particular educational relevance for helping an already supportive audience develop a fuller appreciation of the animal’s abilities and a more personal connection to the species.

The conservation relevance of this approach lies in the opportunity to connect interest in an individual animal with understanding of its species. Bauer and colleagues [60] describe how knowledge of marine mammal cognition can inform conservation by clarifying animals’ responses to environmental challenges. Public interpretation offers a complementary opportunity to communicate that behavioral complexity to visitors. For Hawaiian monk seals, ongoing threats and recently reported deaths emphasize the relevance of education about coexistence [2,6,8,17,20,21,22,23,24]. Pairing cognitive-enrichment demonstrations with guidance on avoiding disturbance and reporting seals in distress could give visitors specific ways to act on their interest. Evaluating that progression would connect immediate engagement with practical conservation outcomes.

Exploratory comparisons detected no differences in rating changes by visitor origin or previous park attendance. Although the subgroup sizes limit interpretation, these results support including both local residents and visitors in further evaluation. Direct measures of prior experience with monk seals and connection to the ocean could provide more informative context than home location alone [77]. Our findings offer a starting point for refining public enrichment presentations to raise awareness of Hawaiian monk seals, deepen understanding of their cognitive abilities, and strengthen visitors’ connection to the species. Such experiences have potential value for conservation education, as research suggests that opportunities for learning and emotional engagement can strengthen concern for wildlife and willingness to support its protection [78,79].

### Limitations and Future Directions

This exploratory study provides an initial assessment of visitor responses to public cognitive enrichment involving one seal at one facility. The single-group design captured immediate changes associated with the overall experience but cannot establish causation or separate the contributions of EVE, animal activity, narration, time at the habitat, and repeated measurement. Self-selection, single-item measures, and questionnaire repetition may have influenced responses, while high baseline ratings limited measurable improvement. The large rank-biserial coefficients describe the predominance of increases among responses that changed and should be considered alongside mean changes and unchanged responses.

For endangered species such as the Hawaiian monk seal, an important next step is to examine whether greater understanding and connection translate into lasting conservation engagement. Future studies could assess retention of conservation messages and actions such as following guidance to avoid disturbing wild seals, reporting animals in distress, and supporting recovery efforts. Comparisons across presentation types, animals, and institutions would help identify how public enrichment can connect interest in an individual animal with measurable benefits for species conservation.

## 5. Conclusions

Following a narrated public EVE demonstration involving a Hawaiian monk seal, visitors reported higher immediate ratings of monk seal cognition and connection. The greatest descriptive changes involved perceived problem-solving and personal connection, whereas protection attitudes and endangered-status knowledge were already high before the session. The computer-game question captured recognition of a demonstrated ability.

This exploratory case study raises a promising question rather than providing a definitive test of educational effectiveness: whether cognitive enrichment designed around an animal’s welfare can also help visitors appreciate its abilities and adaptations, and thereby support connection and concern. Future comparisons of conventional and EVE presentations, along with follow-up measures of learning and conservation-related behavior, can establish when and how that possibility is realized.

## Figures and Tables

**Figure 1:**
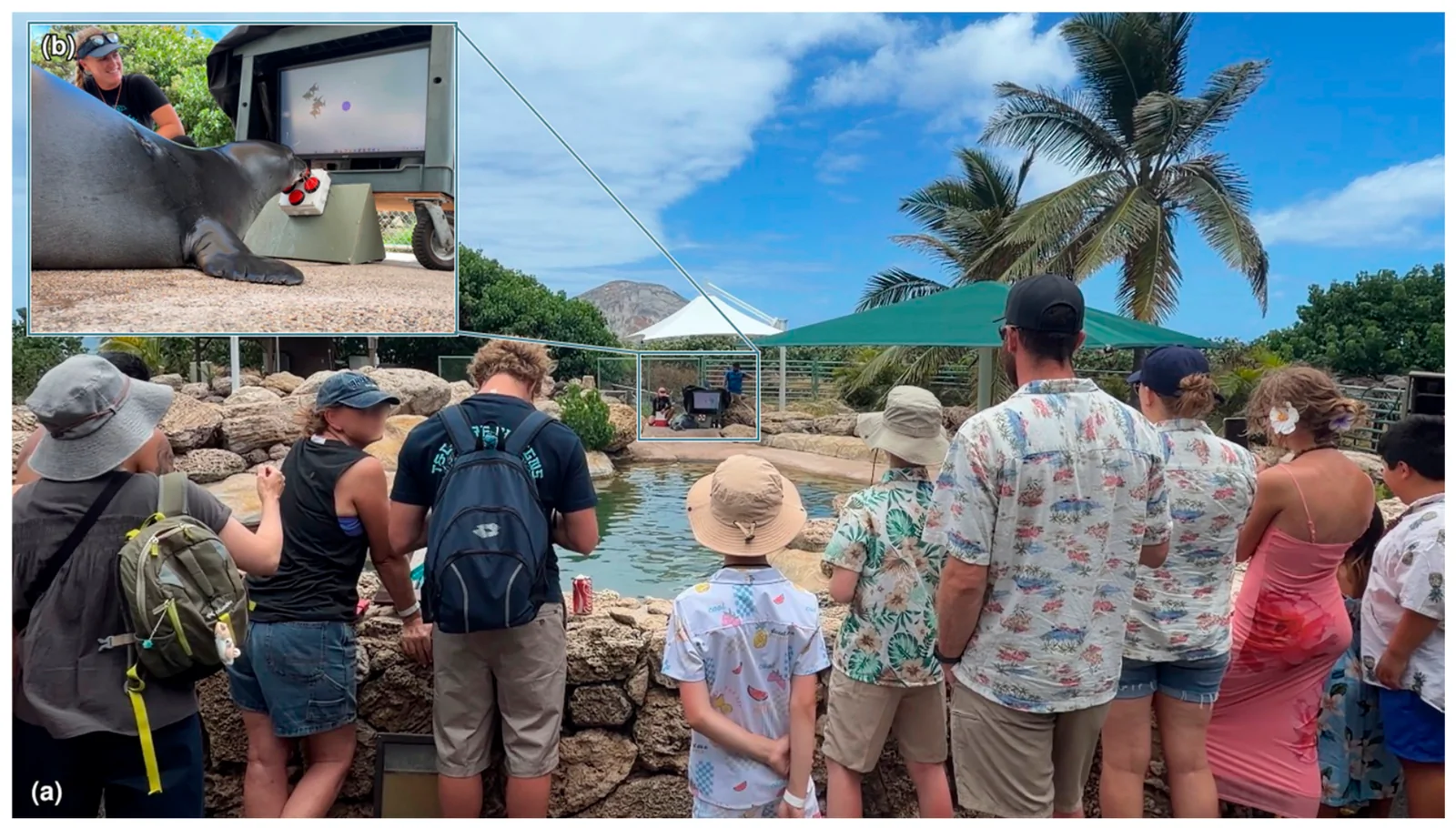
Visitors observing Kekoa during an EVE session (**a**). Kekoa uses his muzzle to press red buttons to move the blue circle in order to contact a moving school of fish (**b**).

**Figure 2:**
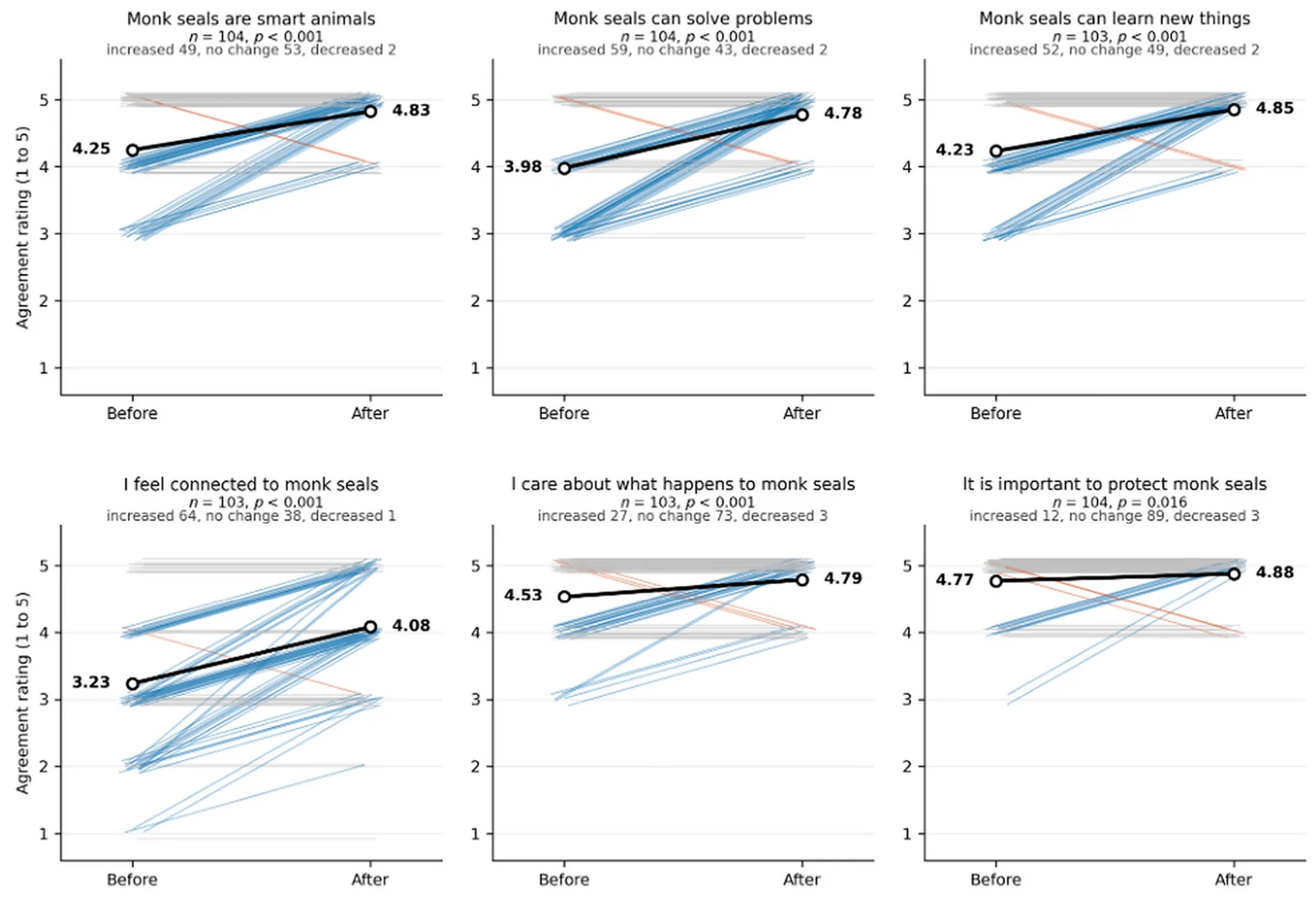
Visitor agreement ratings before and after the enrichment session (*n* = 104 adults). Each thin line connects one participant’s pre- and post- session ratings; blue indicates an increase, orange a decrease, and gray no change. Lines are offset slightly to distinguish identical responses. The heavy black line connects the group means. Each panel also reports the item-specific sample size, the *p* value from a two-sided Wilcoxon signed-rank test, and the numbers of participants whose ratings increased, remained unchanged, or decreased.

**Table 1:** Questionnaire records received and the basis on which each was retained or removed.

Sample selection	Basis	n
Questionnaire records received	All Part 1 submissions, plus Part 2 submissions with no corresponding	146
Excluded: consent declined	Participant selected the non-consent option	2
Excluded: code recorded in one wave only	Part 2 submitted with no matching Part 1 record	2
Excluded: second questionnaire not completed	Questionnaire accepted but the reverse page was left blank owing to a language barrier	2
Matched pre- and post-		140
session records		
Excluded: under 18 years by stated age	Checked box for under 18	18
Excluded: under 18 years by researcher observation	Participant was observed filling out questionnaire on behalf of child	13
Excluded: protocol deviation	One completed Part 1 after the session, two completed both parts before the session was held, and two had observed an EVE session with a different animal before enrolling	5
Analytic sample	Adults with matched pre- and post-session responses	104

**Table 2:** Visitor ratings of monk seal cognition and conservation before and after the public session.

Item	*n*	Before Mdn (IQR)	After Mdn (IQR)	Before M (SD)	After M (SD)	Mean Δ [95% CI]	Up/ Same/ Down	*p* (adj.); *r_b_* [95% CI]; *ρ*
Monk seals are smart animals	104	4 (4–5)	5 (5–5)	4.25 (0.75)	4.83 (0.37)	+0.58 [+0.44, +0.73]	49/53/2	<0.001 (<0.001) *r_b_* = 0.94 [0.84, 1.00] *ρ* = 0.30
Monk seals can solve problems	104	4 (3–5)	5 (5–5)	3.99 (0.84)	4.79 (0.43)	+0.80 [+0.63, +0.96]	59/43/2	<0.001 (<0.001) *r_b_* = 0.96 [0.90, 1.00] *ρ* = 0.25
Monk seals can learn new things	103	4 (4–5)	5 (5–5)	4.24 (0.74)	4.86 (0.35)	+0.61 [+0.48, +0.76]	52/49/2	<0.001 (<0.001) *r_b_* = 0.94 [0.85, 1.00] *ρ* = 0.25
I feel connected to monk seals	103	3 (3–4)	4 (4–5)	3.25 (1.07)	4.10 (0.90)	+0.84 [+0.69, +1.00]	64/38/1	<0.001 (<0.001) *r_b_* = 0.98 [0.93, 1.00] *ρ* = 0.66
I care about what happens to monk seals	103	5 (4–5)	5 (5–5)	4.54 (0.61)	4.79 (0.41)	+0.24 [+0.15, +0.35]	27/73/3	<0.001 (<0.001) *r_b_* = 0.81 [0.57, 1.00] *ρ* = 0.51
It is important to protect monk seals	104	5 (5–5)	5 (5–5)	4.77 (0.47)	4.88 (0.33)	+0.11 [+0.03, +0.19]	12/89/3	0.016 (0.132) *r_b_* = 0.65 [0.20, 1.00] *ρ* = 0.50

Note. n = number of paired responses; Mdn = median; IQR = interquartile range; M = mean; SD = standard deviation; CI = confidence interval. Mean Δ = mean paired change (after−before). Up/same/down = numbers of participants whose ratings increased, were unchanged, or decreased. *p* values are from two-sided paired Wilcoxon signed-rank tests; adjusted *p* values use the Bonferroni correction across eight comparisons. *r_b_* = matched-pairs rank-biserial correlation, calculated from nonzero paired differences; unchanged responses are excluded. *ρ* = Spearman correlation between before and after ratings. Confidence intervals are 95% percentile bootstrap intervals.

## Data Availability

Coded data is available at the request of the corresponding author.
